# Supplementary material for: Enhancement of Dissolving Capacity and Reducing Gastric Mucosa Irritation by Complex Formation of Resibufogenin with β-Cyclodextrin or 2-Hydroxypropyl-β-cyclodextrin
Source: Molecules. 2022 May 17;27(10):3213. doi: 10.3390/molecules27103213 (PMC9146005; doi:10.3390/molecules27103213)
Supplement: Supplementary file 1 [file molecules-27-03213-s001.zip › molecules-1682103-supplementary.pptx]

## Slide 1
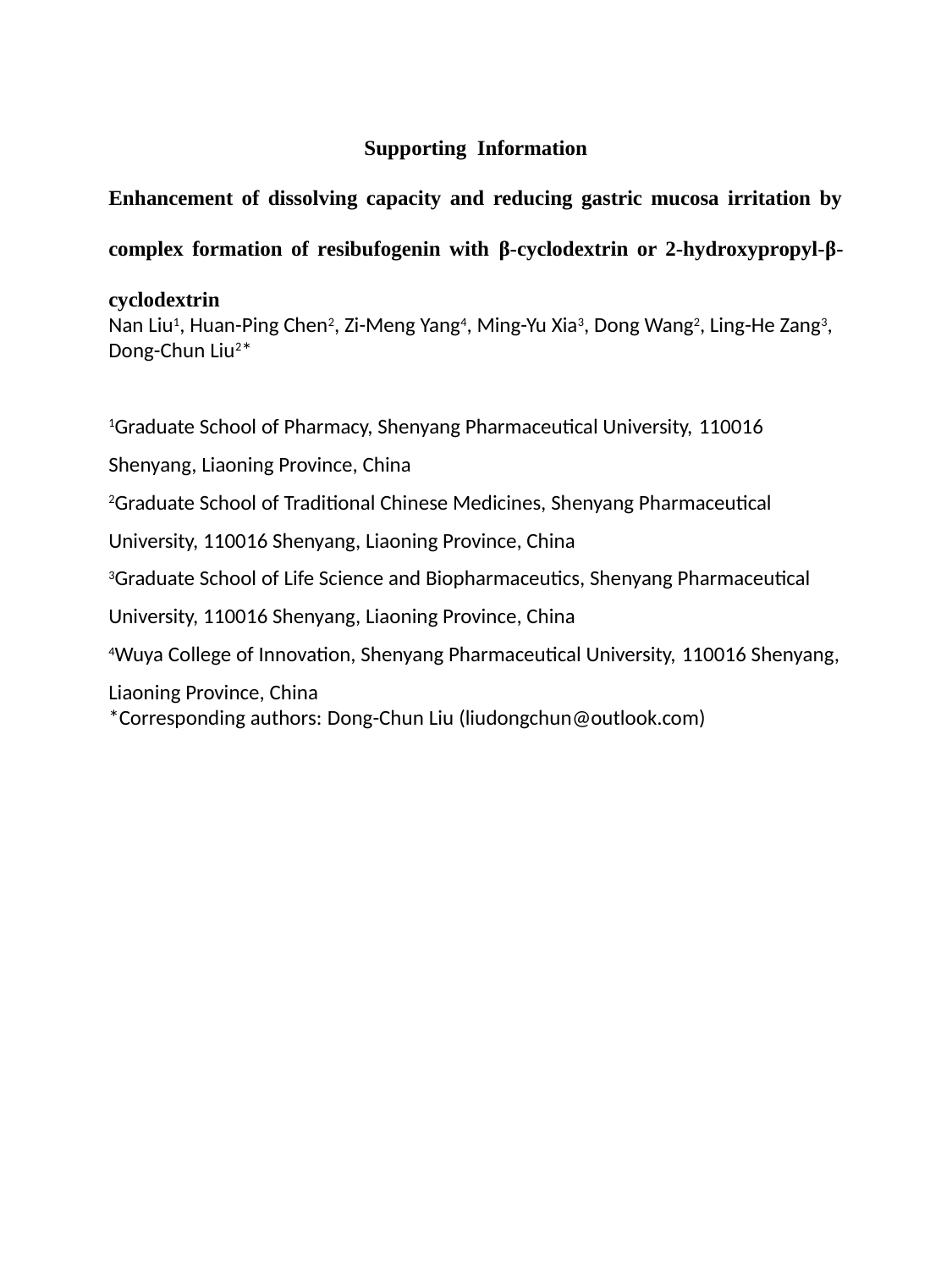

Supporting Information
Enhancement of dissolving capacity and reducing gastric mucosa irritation by complex formation of resibufogenin with β-cyclodextrin or 2-hydroxypropyl-β-cyclodextrin
Nan Liu1, Huan-Ping Chen2, Zi-Meng Yang4, Ming-Yu Xia3, Dong Wang2, Ling-He Zang3, Dong-Chun Liu2*
1Graduate School of Pharmacy, Shenyang Pharmaceutical University, 110016 Shenyang, Liaoning Province, China
2Graduate School of Traditional Chinese Medicines, Shenyang Pharmaceutical University, 110016 Shenyang, Liaoning Province, China
3Graduate School of Life Science and Biopharmaceutics, Shenyang Pharmaceutical University, 110016 Shenyang, Liaoning Province, China
4Wuya College of Innovation, Shenyang Pharmaceutical University, 110016 Shenyang, Liaoning Province, China
*Corresponding authors: Dong-Chun Liu (liudongchun@outlook.com)

## Slide 2
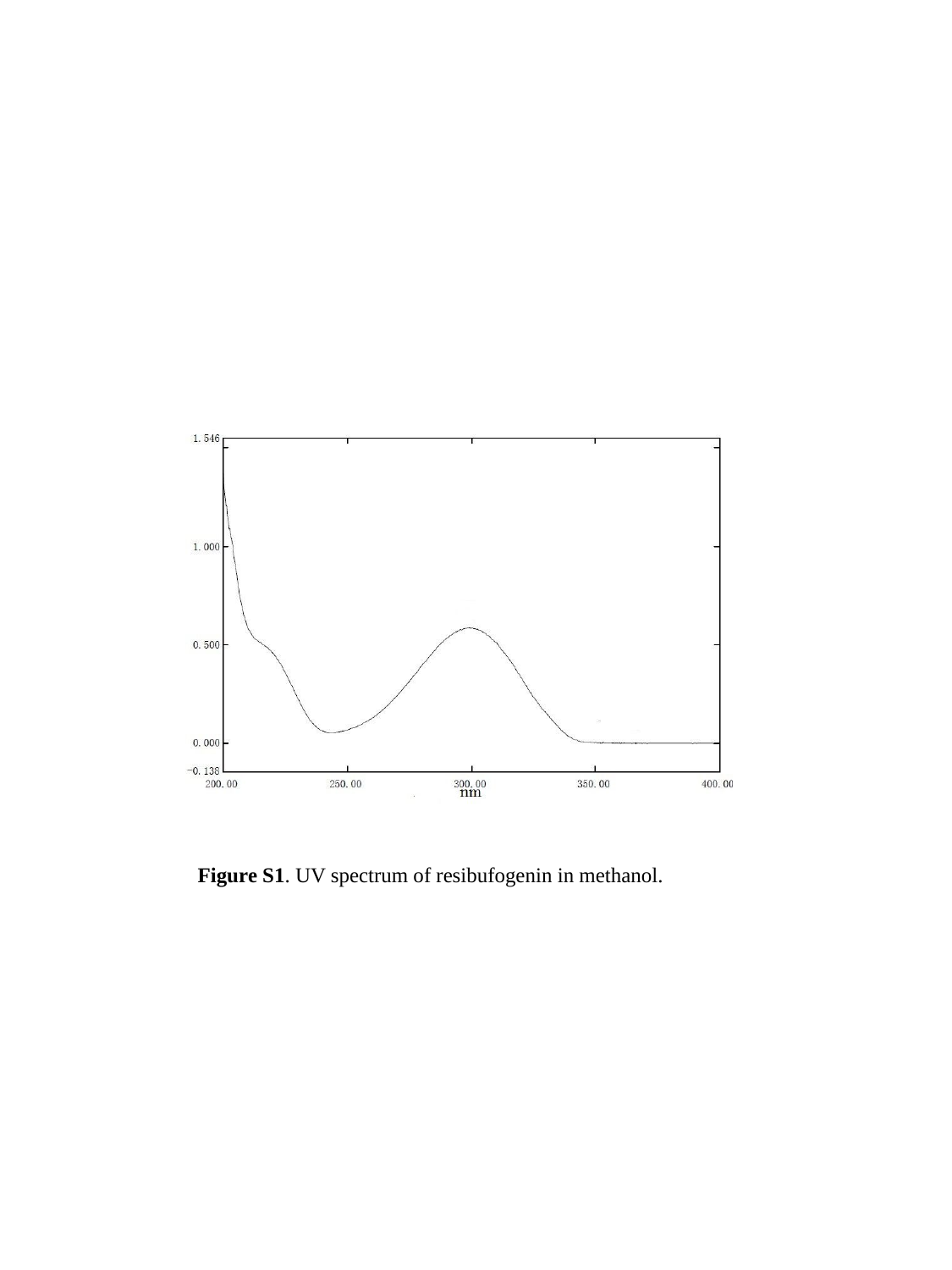

Figure S1. UV spectrum of resibufogenin in methanol.

## Slide 3
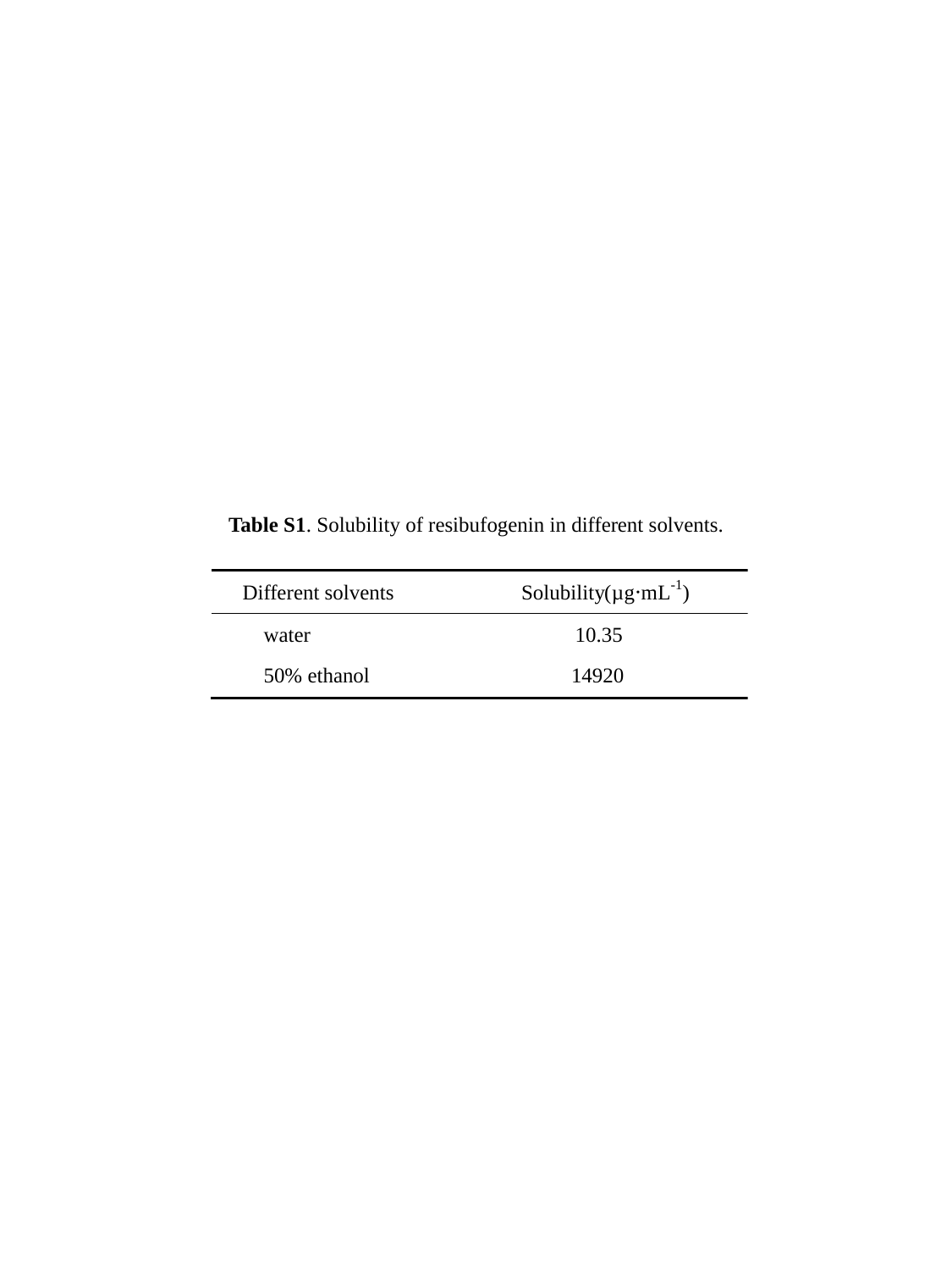

Table S1. Solubility of resibufogenin in different solvents.

## Slide 4
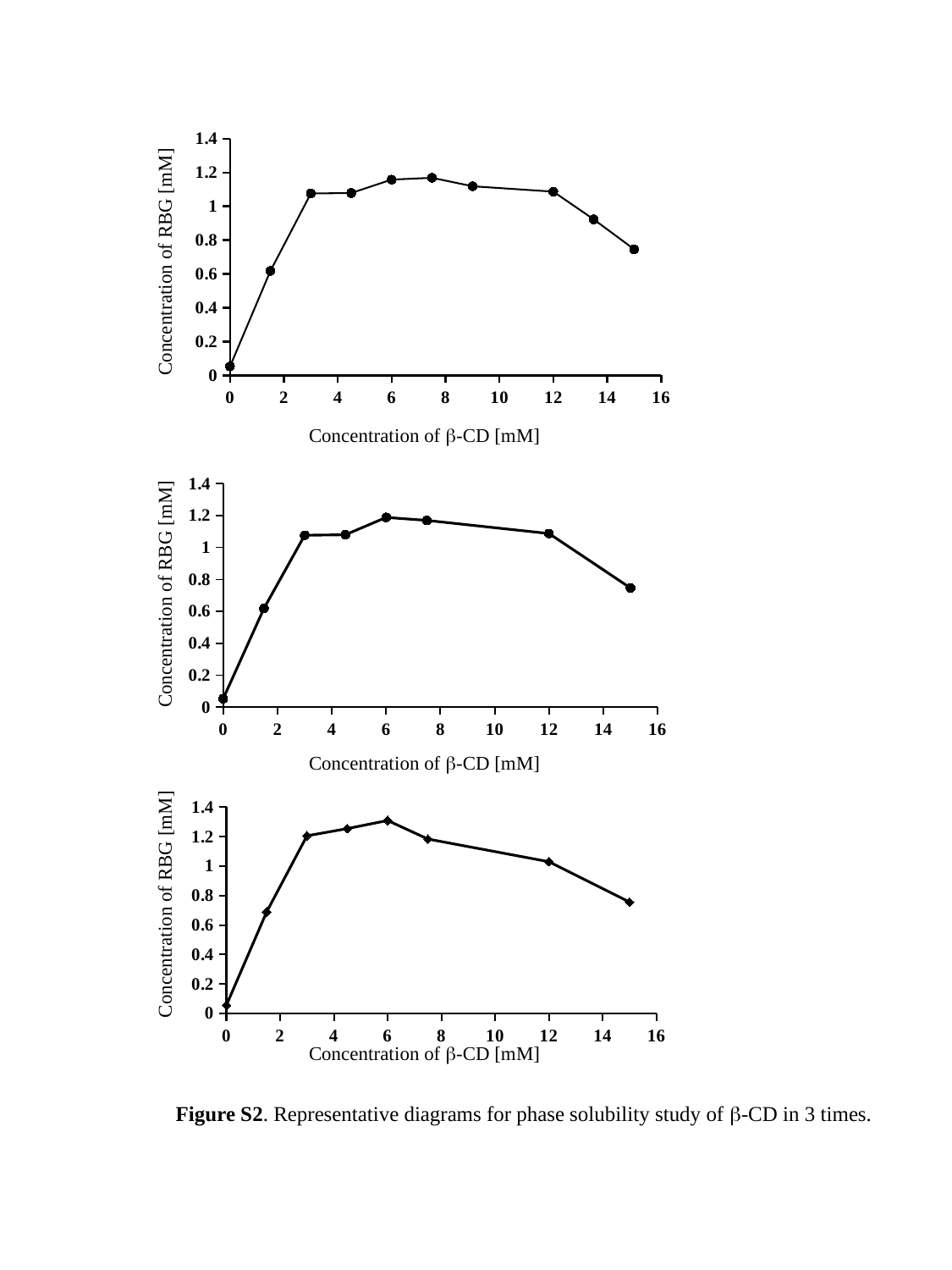

### Chart
| Category | |
|---|---|Concentration of RBG [mM]
Concentration of b-CD [mM]
### Chart
| Category | |
|---|---|Concentration of RBG [mM]
Concentration of b-CD [mM]
### Chart
| Category | RBG浓度（mmol/L） |
|---|---|Concentration of RBG [mM]
Concentration of b-CD [mM]
Figure S2. Representative diagrams for phase solubility study of b-CD in 3 times.

## Slide 5
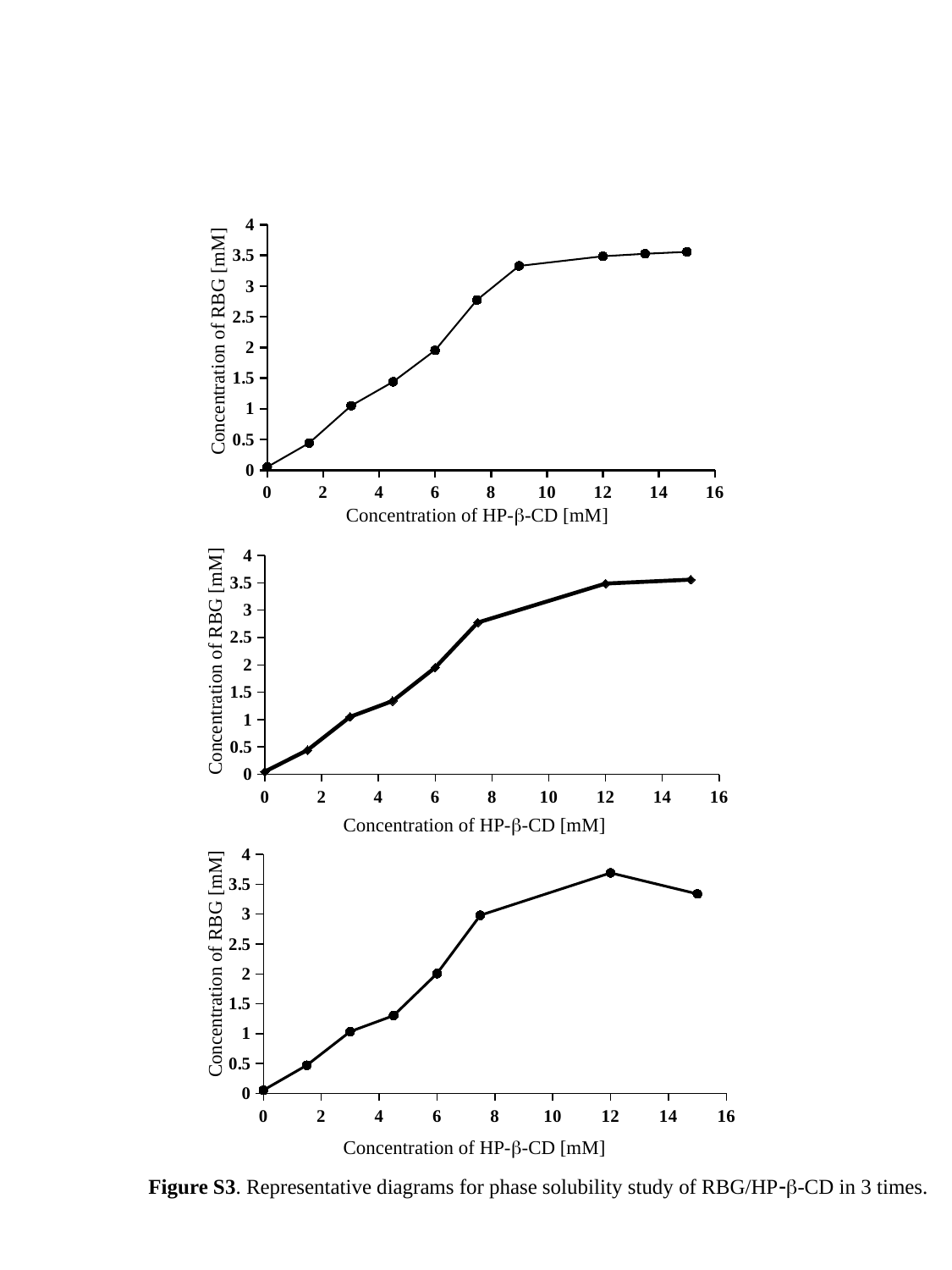

### Chart
| Category | |
|---|---|Concentration of RBG [mM]
Concentration of HP-b-CD [mM]
### Chart
| Category | RBG浓度（mmol/L） |
|---|---|Concentration of RBG [mM]
Concentration of HP-b-CD [mM]
### Chart
| Category | RBG浓度（mmol/L） |
|---|---|Concentration of RBG [mM]
Concentration of HP-b-CD [mM]
Figure S3. Representative diagrams for phase solubility study of RBG/HP-b-CD in 3 times.

## Slide 6
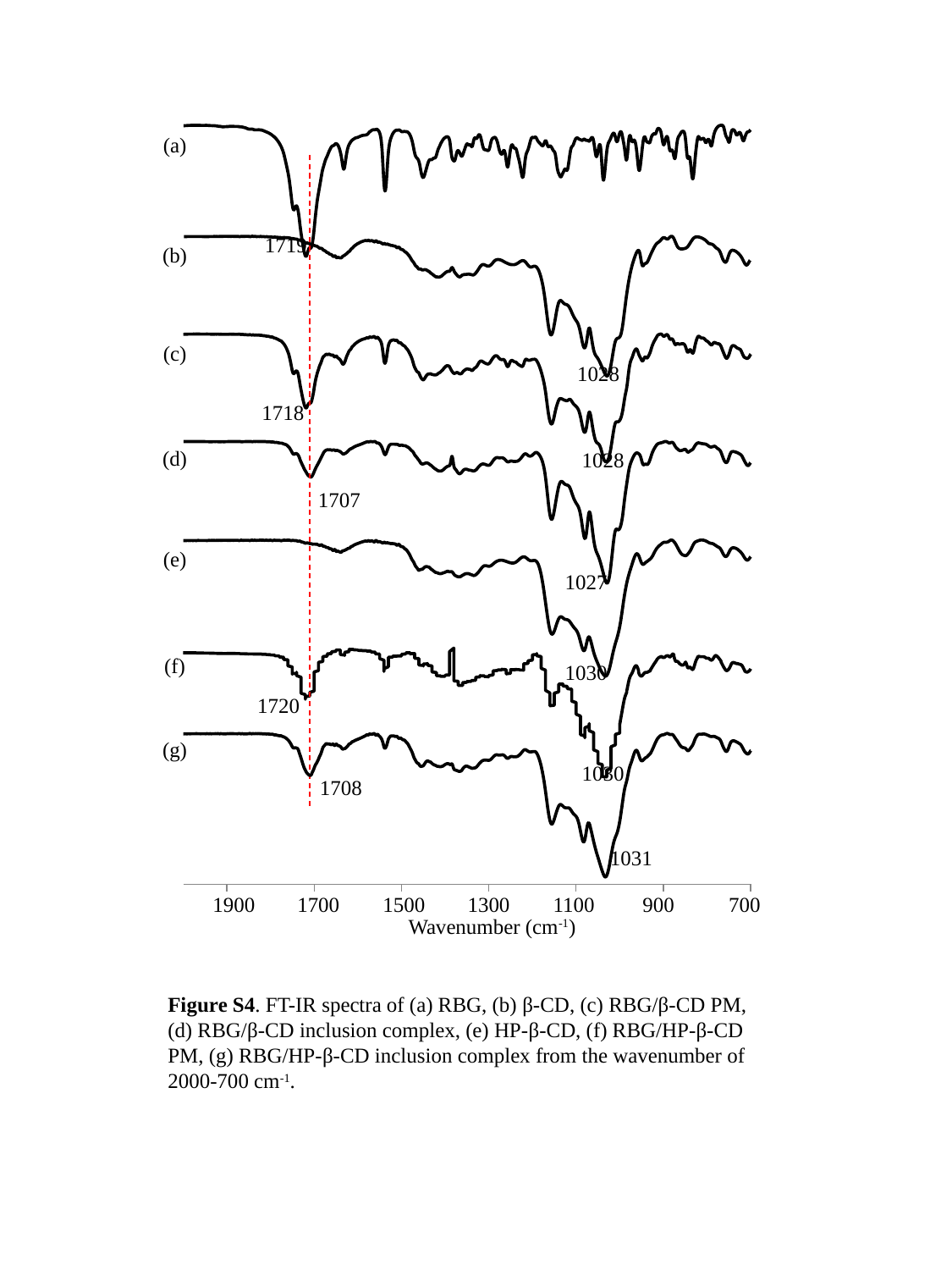

### Chart
| Category | |
|---|---|(a)
### Chart
| Category | |
|---|---|1719
(b)
### Chart
| Category | |
|---|---|(c)
1028
1718
### Chart
| Category | |
|---|---|(d)
1028
1707
### Chart
| Category | |
|---|---|(e)
1027
### Chart
| Category | |
|---|---|(f)
1030
1720
### Chart
| Category | |
|---|---|(g)
1030
1708
1031
1900
1700
1500
1300
1100
900
700
Wavenumber (cm-1)
Figure S4. FT-IR spectra of (a) RBG, (b) β-CD, (c) RBG/β-CD PM, (d) RBG/β-CD inclusion complex, (e) HP-β-CD, (f) RBG/HP-β-CD PM, (g) RBG/HP-β-CD inclusion complex from the wavenumber of 2000-700 cm-1.

## Slide 7
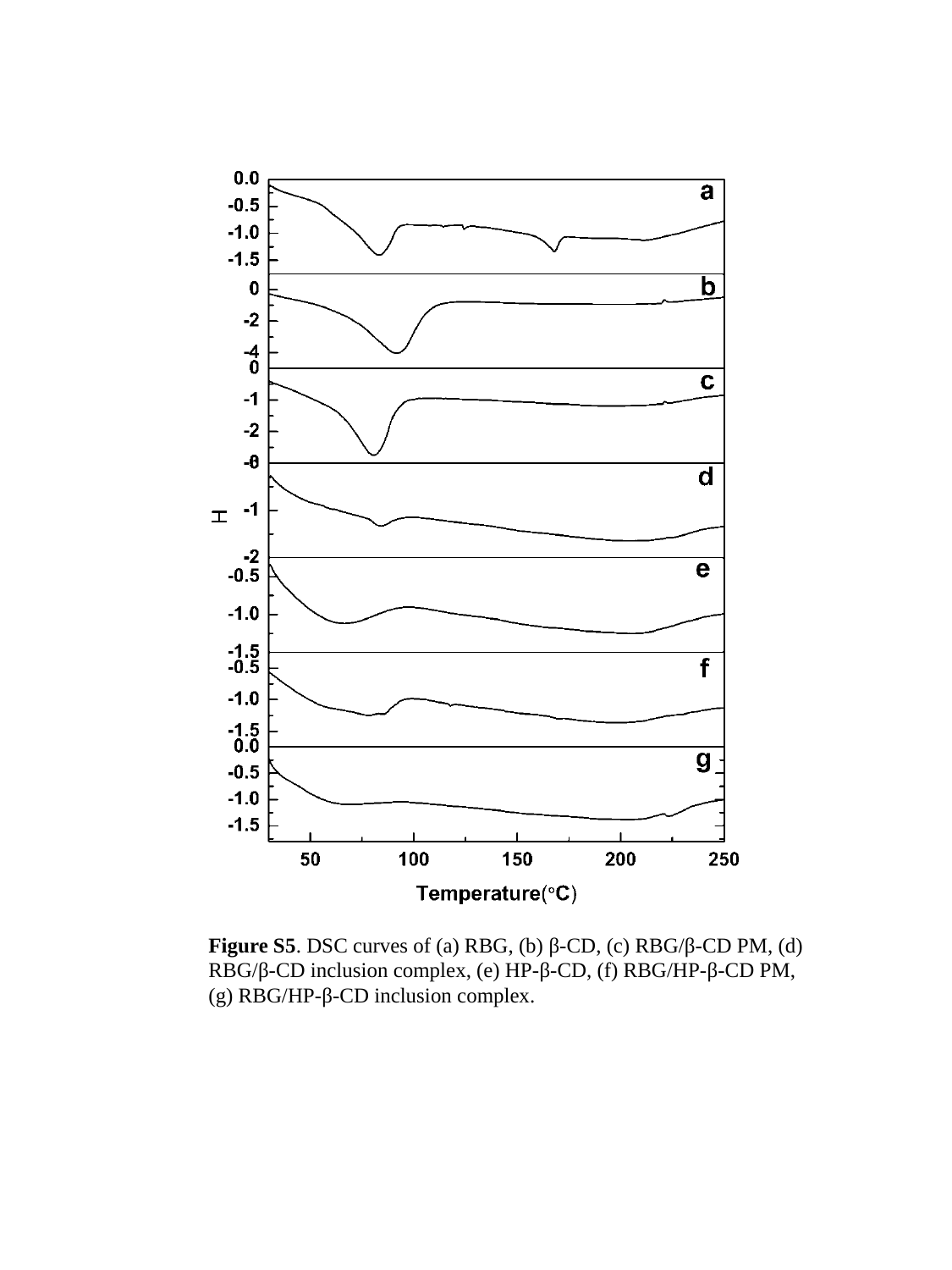

Figure S5. DSC curves of (a) RBG, (b) β-CD, (c) RBG/β-CD PM, (d) RBG/β-CD inclusion complex, (e) HP-β-CD, (f) RBG/HP-β-CD PM, (g) RBG/HP-β-CD inclusion complex.
